# Important role of microglia in HIV-1 associated neurocognitive disorders and the molecular pathways implicated in its pathogenesis

**DOI:** 10.1080/07853890.2020.1814962

**Published:** 2020-09-17

**Authors:** A. Borrajo, C. Spuch, M. A. Penedo, J. M. Olivares, R. C. Agís-Balboa

**Affiliations:** a Department of Microbiology and Parasitology, Faculty of Pharmacy, Complutense University of Madrid, Madrid, Spain; b Department of Experimental Medicine and Surgery, University of Rome Tor Vergata, Roma, Italy; c Department of Psychiatry, Área Sanitaria de Vigo, Vigo, Spain; d Translational Neuroscience Group, Galicia Sur Health Research Institute (IIS Galicia Sur)-Área Sanitaria de Vigo, SERGAS-UVigo, CIBERSAM, Vigo, Spain

**Keywords:** Microglia, Human immunodeficiency virus type 1 (HIV-1), HIV-1-associated neurocognitive disorders (HAND), Reactive oxygen species (ROS), Anti-retroviral therapy (ART), Chemokines (or chemotactic cytokines)

## Abstract

The development of effective combined anti-retroviral therapy (cART) led to a significant reduction in the death rate associated with human immunodeficiency virus type 1 (HIV-1) infection. However, recent studies indicate that considerably more than 50% of all HIV-1 infected patients develop HIV-1-associated neurocognitive disorder (HAND). Microglia are the foremost cells infected by HIV-1 in the central nervous system (CNS), and so, are also likely to contribute to the neurotoxicity observed in HAND. The activation of microglia induces the release of pro-inflammatory markers and altered secretion of cytokines, chemokines, secondary messengers, and reactive oxygen species (ROS) which activate signalling pathways that initiate neuroinflammation. In turn, ROS and inflammation also play critical roles in HAND. However, more efforts are required to understand the physiology of microglia and the processes involved in their activation in order to better understand the how HIV-1-infected microglia are involved in the development of HAND. In this review, we summarize the current state of knowledge about the involvement of oxidative stress mechanisms and role of HIV-induced ROS in the development of HAND. We also examine the academic literature regarding crucial HIV-1 pathogenicity factors implicated in neurotoxicity and inflammation in order to identify molecular pathways that could serve as potential therapeutic targets for treatment of this disease.

**KEY MESSAGES**
- Neuroinflammation and excitotoxicity mechanisms are crucial in the pathogenesis of HAND.
- CNS infiltration by HIV-1 and immune cells through the blood brain barrier is a key process involved in the pathogenicity of HAND.
- Factors including calcium dysregulation and autophagy are the main challenges involved in HAND.

## Graphical Abstract

**Figure:**
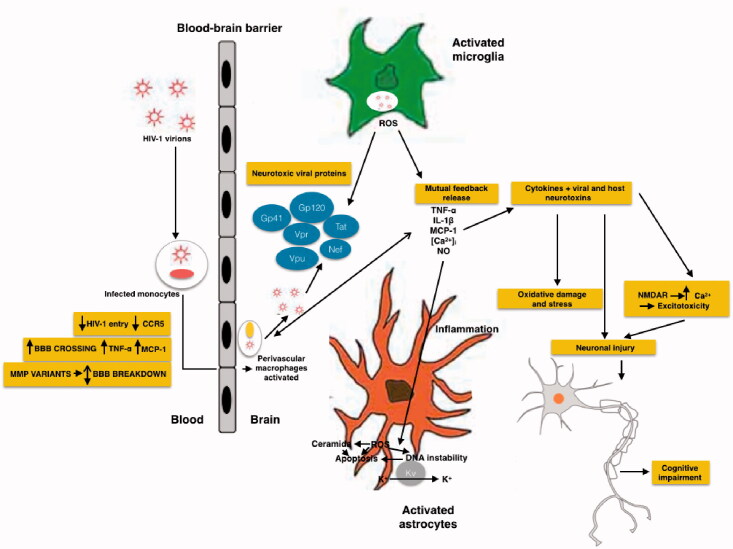

## 1. Introduction

Human immunodeficiency virus 1 (HIV-1) causes a chronic infection, which can lead to severe immunodeficiency. The first major advance made to control the clinical course and progression of this disease came from studies that tested drugs used to suppress HIV replication. This was followed by the evolution and implementation of antiretroviral therapies (ARTs), which can support efficient systemic suppression of HIV replication and has reduced the number of deaths resulting from acquired immune deficiency syndrome (AIDS) [1]. Nonetheless, even though treatment with current ARTs reduces HIV-1 ribonucleic acid (RNA) levels and increases the survival of people living with HIV-1, this virus is still able to evade immune responses *via* several mechanisms, including by establishing a persistent infection within different cell types [2].

Subsequent research then started shifted towards optimising the personalized treatment of individual patients by monitoring factors associated with HIV-1 such as the levels of CD4^+^ helper T cells, plasma HIV RNA, and antiretroviral resistance profiles; this approach has now become standard and is integrated into clinical practice [3]. Finally, the care of HIV + patients was significantly advanced by initiating ART as soon as the patient agrees to commit to this lifelong therapy, regardless of their CD4^+^ T cell count [3]. This recommendation was reinforced by results from the strategic timing of antiretroviral therapy (START) trial, which confirmed the safety of starting ART earlier and its benefits in terms of overall HIV infection outcomes [1,3].

This progress has increased the life expectancy of patients infected with HIV with access to treatment [4], and moreover, because the immune systems of these individuals is reconstituted, their infection with opportunistic diseases has also become infrequent. However, cognitive disorders related to HIV still remain [5]. A HIV-1 infection status often causes neurological symptoms including cognitive impairment and motor disturbances referred to as HIV-1-associated neurocognitive disorder (HAND) [6,7]. In general, HAND is characterized by a combination of virus-related neurological disorders and neuronal-tissue inflammation [6,8]. The use of ARTs able to penetrate the blood-brain barrier (BBB) may reduce the incidence of these complications, but not all anti-HIV drugs are able to effectively cross this barrier [8,9] meaning that viral reservoirs still persist, which contribute to HAND [10,11].

Thus, the clinical symptoms of HAND remain an important problem for chronic HIV-infected patients, particularly among children or those whose adherence to ART is low [10]. Additionally, the symptoms of HAND are becoming increasingly common among the advanced-age HIV population because this risk factor is associated with functional deterioration and disability [12]. Another important risk factor is a low CD4^+^ T-cell count in the presence of the virus, an increased plasma viral load, HCV-coinfection, and metabolic comorbidities in HIV-infected patients with neurocognitive disorders [6,8]. In fact, several challenges remain in the drive towards a cure for HIV-1, including defining and locating every potential HIV-1 reservoir, determining their order of importance, and size, and developing ways to measure the effectiveness of HIV-1 eradication strategies. This information will be key to informing future studies and ensuring that any new approach considers all the variables that could impact treatment outcomes [13].

## 2. Hiv-associated neurocognitive disorders

### 2.1. Classification and diagnosis of HIV-associated neurocognitive disorder phenotypes

Many diagnostic proposals have been suggested for use during the diagnosis of HAND [14,15], although the Frascati criteria [16] are the most universally used nosology and are considered the gold standard in HIV research. This scheme identifies three HAND severity levels: asymptomatic neurocognitive impairments (ANIs), mild neurocognitive disorders (MND), and HIV-associated dementia (HAD) [17]. ANI is typified by cognitive impairment and involves at least two cognitive domains that do not interfere with everyday function. MND represents a cognitive impairment that involves at least two cognitive domains that produce at least a mild interference in daily function. Finally, HAD is related to marked cognitive impairment which involves at least two cognitive domains that substantially interfere with, and are markedly incompatible with, daily functioning [17] (Table 1).

**Table 1.** The role of viral regulatory proteins in HAND.

Viral gene	HIV proteins	Localization	Action	Mechanism of action	References
Structural	gp120	Membrane of infected cells (envelop protein)	Direct and indirect damage	Meddles in N-methyl-D-aspartate receptor; produces oxidative stress; provokes activation of macrophages, microglia and astrocytes; induces release of cytokines and blocks glutamate uptake	Galicia et al. 2002; Nath 2002; Chen et al. 2011; Kovalevich and Langford 2012; Zhou and Saksena 2013; Ivanov et al. 2016; Scutari et al. 2017
Structural	gp41	Membrane of infected cells (envelop protein)	Direct and indirect damage	Induces cytokines and produces nitric oxide by iNOS	Nath 2002; Kovalevich and Langford 2012; Mastrantonio et al. 2016
Regulatory	Nef	Surface of infected cells	Direct damage	Inhibits K channels	Nath 2002; Mangino et al. 2015; Scutari et al. 2017
Regulatory	Tat	Secreted into extracellular medium	Direct and indirect damage	Enhances intracellular calcium; produces reactive oxygen intermediates; actives cells of inmune system; induces release of cytokines and chemokines and directs excitation of neurons	Haughey et al. 2001; Galicia et al. 2002; Nath 2002; Kanmogne et al. 2005; Ivanov et al. 2016; Scutari et al. 2017
Regulatory	Vpr	CSF of HAND patients	Direct damage	Forms ion channels across cell membrane	Galicia et al. 2002; Nath 2002; Kanmogne et al. 2005 Ivanov et al. 2016; Scutari et al. 2017
Regulatory	Vpu	Unknown	Direct damage	Increases ion channel activity	Eckstein et al. 2001; Nath 2002; Varthakavi et al. 2003; Hsu et al. 2004; Kovalevich and Langford 2012

### 2.2. Pathology of neuro AIDS

The pathogenesis of HAND is complex and multidimensional and recent studies have suggested that its pathophysiology is more likely associated with functional alterations in neurons. The controversial question remains whether HIV can infect neurons, even at low levels. Many studies dating back to the 1980s and 90 s reported that HIV could indeed infect neurons in the brain *in vivo*. Furthermore, previous work using *in situ* polymerase chain reaction (PCR) and immunohistochemistry showed the presence of HIV genetic material and antigens in neurons, respectively [18]. Additional research which isolated neurons from the post-mortem brain tissues of HIV-infected patients by laser capture microdissection (LCM), described the presence of pro-viral HIV deoxyribonucleic acid (DNA) in neurons by PCR [19]. Other previous work employed hyperbranched multi-displacement techniques for whole-gene amplification *via* PCR and identified HIV DNA in single neurons collected by LCM from brain tissue obtained in autopsies [20]. Moreover, *in vitro* studies indicate that human neuronal cell lines could be infected with HIV [21], although the verification of a pathologically significant infection in adult human neurons *in vivo* is still lacking.

**Figure 1.**
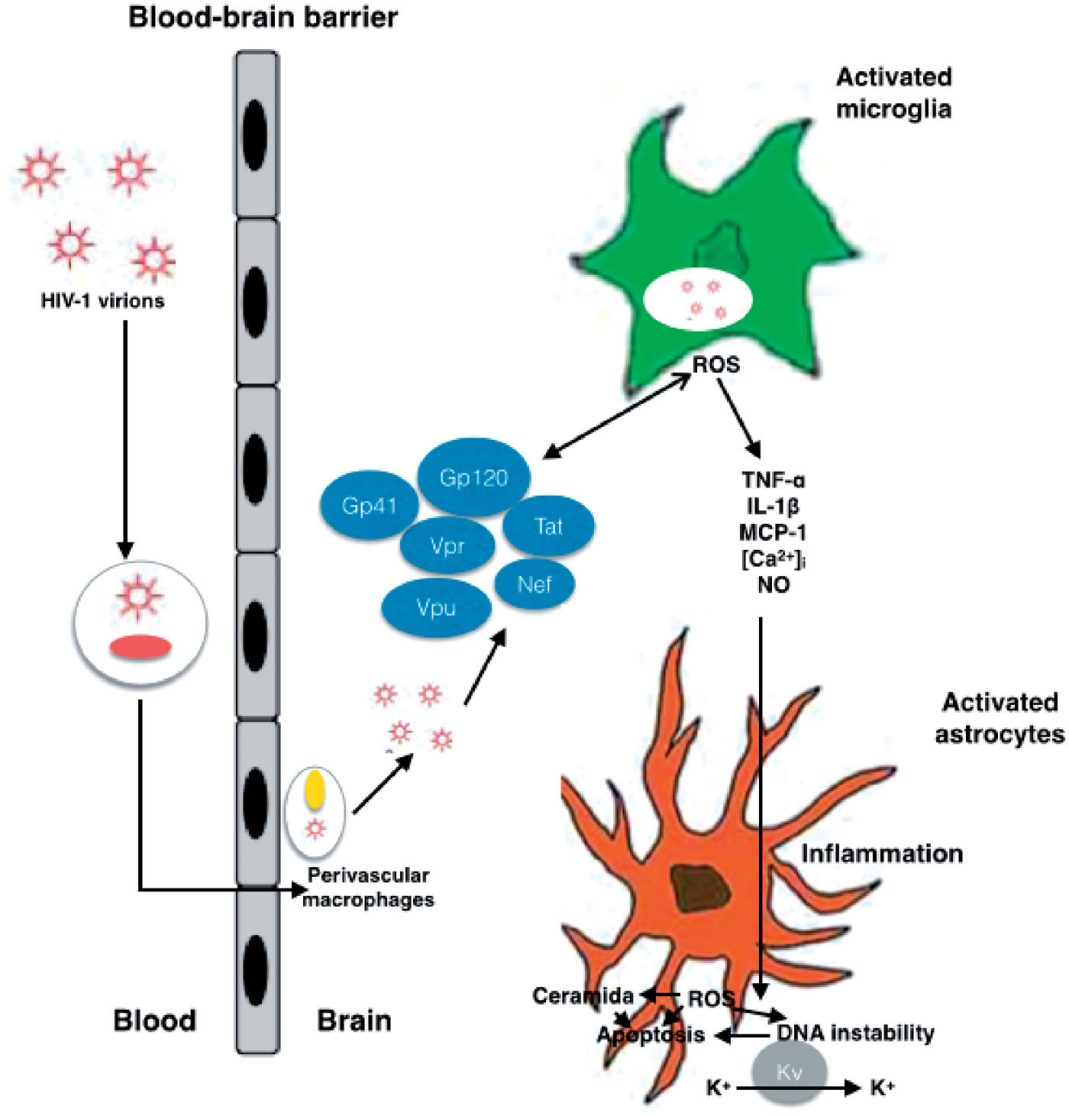
Schematic representation of mechanisms of HIV neurotoxicity. The Gp41, Gp120, Tat, Vpr, Vpu and Nef viral proteins, that circulate in the blood produce enhanced ROS production, results in alteration of BBB. Different process occurs during the neurotoxicity like enhanced oxidation of DNA nucleic bases, genomic instability, aggregation of ceramide, stimulation of A-type transient outward K + currents by Kv channels, and production of proinflammatory cytokines.

HIV can be detected in the central nervous system (CNS) of infected adults, paediatric patients, and developing foetal brain [22]. However, studies done in adult HIV-infected brain tissues and paediatric HIV patients have failed to find conclusive evidence of whether HIV infects neurons [23]. Nonetheless, HIV is thought to enter the CNS early during infection, primarily through infected lymphocytes and monocytes crossing the BBB [24]. Viral proteins, such as transactivator of transcription (Tat), viral protein R (Vpr), and Glycoprotein 120 (gp120), cause neuronal injury and/or apoptosis *via* tumour necrosis factor alpha (TNF-α), interleukin-6 (IL-6), and interleukin-1 (IL-1), increase intracellular calcium ion load and reactive oxygen species (ROS) production [17] (Figure 1 and Table 2) by activating macrophages, microglia, and astrocytes by binding to the α-chemokine receptor, C-X-C 4 (CXCR4) and C–C chemokine receptor 5 (CCR5) [25].

**Table 2.** Typification by cognitive impairment and recommendations for Monitoring Patients With HAND.

Stage of HAND	Neurocognitive impairment and functional status	Clinical recommendations	References
ANI	Involves two cognitive domains that do not interfere with everyday function	Monitored initially at six months and re-evaluated at month twelve	(Bouwman et al. 1998; Antinori et al. 2007; Mind Exchange Working Group 2013; Scutari et al. 2017)
MND	Involves, at least, two cognitive domains that produce, at least, mild interference in daily function	Monitored clinically initially at months three and six, then semi-annually until a plateau of response have been observed	(Antinori et al. 2007; Cysique et al. 2009; Heaton et al. 2011; Mind Exchange Working Group 2013; Scutari et al. 2017)
HAD	Involves, at least, two cognitive domains that substantially interferes with daily functioning	Periodically reassessed, perhaps as frequently as monthly if practical	(Cysique et al. 2006; Antinori et al. 2007; Heaton et al. 2011; Mind Exchange Working Group 2013; Scutari et al. 2017)

**Figure 2.**
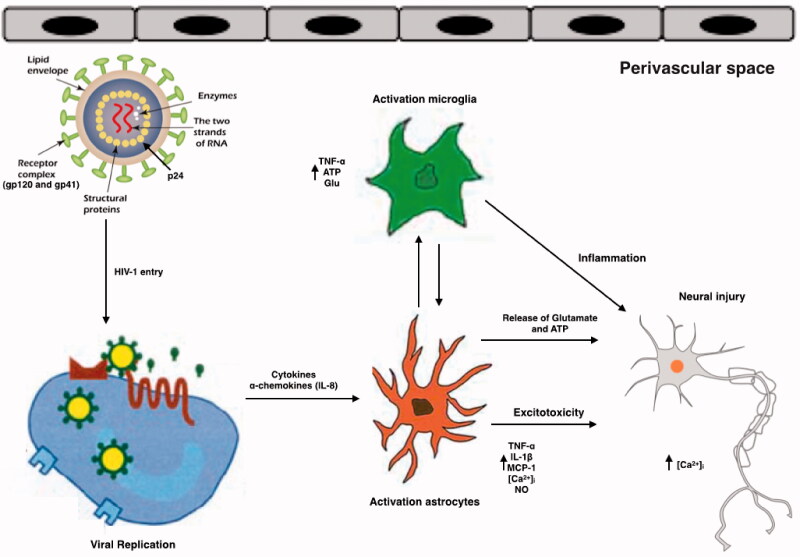
Schematic representation of the effects of HIV and its viral proteins on the cells of the CNS of HAND patients. HIV enters into the brain, especially perivascular space (the main site for viral replication), within infected macrophages or as free virions or viral particles. HIV infects and activates macrophages, astrocytes and microglia in the perivascular space, the main site for viral replication. Macrophages, and astrocytes and activated microglia contribute to the release of proinflammatory cytokines and chemokines, which provoke additional influx of immune cells and mediate neuronal damage. Conversely, some inflammatory mediators can also promote neuronal survival. HIV induces activated astrocytes which secrete proinflammatory cytokines, chemokines and glutamate. Together with viral proteins and HIV-induced chemokines, these substances overstimulate NMDA receptors, causing excitotoxicity. All the events caused by HIV proteins, excitotoxicity, and inflammation lead to axonal injury and neuronal apoptosis.

CCR5 is a crucial co-receptor that allows HIV to enter monocytes and microglia. Mutated CCR5 affects HIV disease progression and indeed, prior to the combined anti-retroviral therapy (cART) era, caused neurocognitive impairment [26]. HIV entry involves an initial interaction between gp120 and the host CD4 receptor after which, gp120 binds to the CCR5 co-receptor on the host cell; CCR5 mediates HIV infection of macrophages and microglia. Interestingly, a mutation in CCR5 produces a truncated CCR5 protein known as the CCR5D 32 variant, and heterozygosity for CCR5D 32 is associated with a reduced risk of impaired cognitive function in patients with HAND [26]. Infected/activated macrophages and microglia release substances such as adenosine triphosphate (ATP), arachidonate, and excitatory amino acids including glutamate, quinolinate, and cysteine, while infected astrocytes release glutamate and nitric oxide (NO) radicals [27] (Figure 2). In addition, CD4^+^ memory T cells, that are usually infected by primary HIV-1 infection, act as major latent HIV reservoirs during inflammatory events, even when ART is used.

## 3. Microglia are crucial cells in the pathogenesis of HIV-associated neurocognitive disorder

Phagocytic cells in the CNS that later became known as microglia were discovered by Franz Nissl, Alois Alzheimer, Ramón y Cajal, and Pío del Río-Hortega during the 19th and 20th centuries [28]. Microglia, which can migrate, proliferate, and phagocytose, constitute some 10–15% of all brain cells which can be distinguished from neurons and other glial cells present in the CNS by their morphological characteristics [28]. In the context of neurodegenerative diseases, microglial cells play essential roles in the clearance of Beta amyloid [29] and Tau protiens [30]—whose accumulation significantly correlates with the presentation of neurocognitive impairments. Microglia express receptors for various neurotransmitters as well as for innate immunity ligands, including pattern-recognition receptors such as toll-like receptors [31].

Peripheral system macrophages are a microglial cell type that presents antigens and secretes cytokines [32–36] involved in the physiological processes implemented to combat pathogens and repair tissues. Cytokines are low molecular weight proteins that are generally classified as pro- or anti-inflammatory. While pro-inflammatory cytokines can elicit a sustained immune response, anti-inflammatory cytokines downregulate immune responses by binding to receptors expressed in the microglia to initiate an autocrine signalling process. Cytokines have several effects on the function of the CNS such as promoting the growth and proliferation of astrocytes and oligodendrocytes [33] and modulating the release of neurotransmitters [31]. Recent studies have shown low levels of pro-inflammatory cytokines are expressed in the healthy brain, while they are expressed in high levels in brain and cerebrospinal fluid (CSF) samples from patients with Parkinson disease, Alzheimer disease [37,38], or other psychiatric diseases like schizophrenia [39].

In the adult brain, most microglia are in a “resting” (quiescent) state and typically have a branched morphology. In 1996, Kreutzberg [40] proposed a microglia classification system according to their *in vivo* morphology as: branched with small cells and many thin branches (quiescent), or amoeboid with truncated processes (an active status) to facilitate proliferation, migration, and phagocytosis [41]. Quiescent microglia are not static, rather, their branches constantly move. Indeed, studies carried out in mice have shown that microglial cells expand and retract dynamically [41,42]. Microglia activation occurs during the development and remodelling of healthy brain tissue and apoptosis occurs during the early embryonic and postnatal brain development to avoid cell over population [43]. The human brain microglia population is sustained by a continuous slow turnover of cells throughout adult life [44].

One of the most commonly used methods for activating microglia *in vitro*, mimics their *in vivo* activation, by applying lipopolysaccharide endotoxin (LPS). Microglia activation can be either neurotoxic or neurotrophic, and this is highly context dependent. “Acutely activated” microglia (by treating them with toll-like receptor 4 ligand and LPS for 24 h) release pro-inflammatory cytokines and chemokines such as Interleukin 1β (IL-1β) and Macrophage inflammatory protein 1β, and these contribute to a reduced neuronal survival rate. In contrast, “chronic activation” of microglia (LPS exposure for 72 h) induces secretion of anti-inflammatory cytokines and the development of a neuroprotective phenotype [45]. It has also been suggested that microglia become activated *via* a “switching-off” process in which they are stimulated by the presence of damaged neurons, causing them to leave M_0_ and enter M_1_. Finally, *in vitro* it appears that healthy neurons can decrease the microglial response when stimulated by low doses of LPS, resulting in reduced NO and TNF-α levels [17].

Clues obtained in primary human cell cultures indicate that microglia and monocyte-derived macrophages (MDMs) have divergent activation programs [46]. When stimulated with M1-polarizing agents such as Interferon γ (IFNγ), both adult and foetal human microglia and MDMs express elevated levels of CD80 and CCR7, although less so in foetal microglia. Treatment with M2-polarizing agents such as Interleukin-4 leads to significantly increased expression of CD23, CD163, CD206, and CD209 in MDMs; CD209 in adult microglia; and CD209 and CD206 in foetal microglia [46,47].

Four major types of macrophages have been identified in the CNS: meningeal, choroid-plexus, perivascular, and microglia [6,48,49]. Many functional characteristics discern microglia from other macrophages, such as their distinct activation patterns. For instance, adult microglia express qualitatively similar but quantitatively lower levels of several antigenic markers, including CD45, CD11b, HLA-DR, CD14, CD200R, CD16, CD32, CD64, CD86, CD163, CD68, CD4, and Iba-1, compared to macrophages [50]. Hence, through gene profiling and quantitative mass spectrometry analysis of murine cells, high expression levels of a group of transforming growth factor β (TGF-β)-dependent molecular signatures only in microglia when compared to other neuronal and immune cells (including resident macrophages in peripheral tissues) [51].

Microglia carry out different immunomodulatory activities to protect neuronal stability. Indeed, impaired microglial action is linked to the development of several neurological disorders including Alzheimer, Huntington, and Parkinson diseases and such impairment plays an important role in the pathogenesis of HAND by contributing to neurodegenerative events through various mechanisms. Because these cells are resistant to the cytopathic effects of HIV-1, they can sustain the infection for prolonged periods of time [6]. They subsequently become involved in inflammation by releasing HIV proteins, inflammatory cytokines, and neurotoxins, which then induce astrocyte differentiation and apoptosis, as well as the alteration of normal neurogenesis [47]. Thus, microglia play a crucial role in mediating the neurodegeneration process.

Glial cells infected by HIV can induce the production of neurotoxins (the platelet-derived growth factor and QUIN inflammatory mediators), resulting in the release of viral proteins (gp120, Tat, and Vpr) which damage neurons and astrocytes [48] and activate virus replication. *In vitro* studies have shown that viral factors may induce the release of other chemo-attractive factors in HIV-infected glial cells, which then recruit monocytes and microglia, thus amplifying this neuronal damage [6]. Additionally, several cellular factors such as IL-1β, TNF-α, and interferon gamma (IFN-γ) can stimulate and reactivate viral replication in latently infected glial cells [52].

The BBB is a neurovascular unit formed through interactions among astrocytes, pericytes, microglia, and the basement membrane, which protects the fragile neuronal microenvironment from neurotoxic substances [24]. Transport *via* endothelial cells across the BBB is rigorously regulated through interactions with the cells comprising it, including microglia. Thus, the deactivation of microglia *via* the release of cytokines or ROS can adversely influence integrity of the BBB [51]. Another important component of the BBB are neuroectodermal-derived cells, which support the function and metabolism of neurons, ionic CNS homeostasis, scar formation, neuronal synapse status (by taking up neurotransmitters), and tissue repair [6]; these cells also regulate the immune response in the brain [52]. Additionally, another BBB component—astrocytes—can support low-level HIV replication, which allows the virus to persist in the CNS [52] and making it possible to establish a latent infection. Astrocytes can be responsible for increasing the release of intracellular calcium (Ca^2+^) in the brain, resulting in caspase activation and p53 expression [6,52], thus contributing to HAND. Finally, oligodendrocytes reduce myelin synthesis and increase intracellular Ca^2+^ levels and cellular apoptosis in the brain [6,52], and this demyelination in the cerebrum may produce cognitive impairment in HAND.

## 4. The influence of microglia in disrupting and compromising the blood-brain barrier in HIV-associated neurocognitive disorder

As mentioned above, the BBB protects the brain by preventing fatal substances from freely entering the CNS. However, the BBB can be disrupted by several pathological processes [53], which may contribute not only to accelerating infection of the brain by HIV but could also alter CNS function. HIV affects the brain shortly after infection, and studies of CSF, as well as dynamic studies of the BBB with contrast enhanced magnetic resonance imaging, have confirmed abnormalities in the BBB in people infected with HIV [54,55]. In fact, pathological studies of the CNS have identified a range of pathogenic mechanisms for HIV-associated compromise of the BBB, both *in vivo* and *in vitro*. Furthermore, BBB disruption also has implications with respect to ART [56].

A recent study explored the role of BBB disruption in the pathogenesis of HAND in the context of fully suppressive ART. They used dynamic contrast enhanced perfusion MRI to measure capillary permeability as an indicator for BBB integrity and found that the incidence of HAND in these patients was associated with BBB impairment. This work highlighted region-specific rather than global BBB disruption, and no correlation with neuroinflammation blood markers, suggesting that HIV and not systemic inflammation was driving this BBB disturbance. This would mean that the BBB disruption was a consequence of HIV already present in the brain as opposed to HIV impairing the BBB and then causing the brain disease [53]. Another study explored the establishment of abnormal BBB permeability and its relationship to neuropathogenesis during primary HIV infection by evaluating the CSF to serum albumin quotient (QAlb). To date, the QAlb remains the best-known fluid marker for BBB permeability in patients with primary HIV infection. They found after initiating ART during primary HIV infection, QAlb increases in association with neuronal damage, and this increase did not significantly improve during treatment over one year. Thus, they concluded, BBB-associated neuropathogenesis in HIV-infected patients may start during primary infection [52].

HIV-1 causes disruption of endothelial junctions [57] and is thought to invade the brain parenchyma *via* a “Trojan horse” mechanism by diapedesis of infected immune cells that either cross the BBB paracellularly (between cells) or transcellularly (through cells) [56,58]. HIV-1 provokes alterations in the expression of tight junction proteins, likely in a CCL2-dependent manner, thus facilitating access of HIV-infected cells across the BBB. These include (1) the inhibition of tight junction protein expression; (2) a concomitant increase in matrix metallopeptidase 9 (MMP9) caused by Tat which results in tight junction protein cleavage [59]; (3) activation of Ras signaling [60]; (4) an increase in vessel permeability because of the presence of secreted viral surface gp120; (5) increased expression of matrix metallopeptidase 2 (MMP2) and MMP9 in *in vitro* culture supernatants [61]; (6) and decreased expression of tight junction proteins Claudin-5 and laminins caused by oxidative stress [62], all of which contributes to the pathogenesis of HAND. The HIV proteins gp120 and Tat are specifically known to disrupt the BBB. Indeed, recent work has shown increased leakiness of fluorescent tracer compounds into the brains of Tat transgenic mice [63]. *In vitro*, treating endothelial cells with Tat and gp120 decreases several important tight junction proteins, with resultant increases in endothelial monolayer permeability [64,65].

HIV is also thought to enter the brain through infected macrophages, which traverse the vascular endothelium associated with the BBB [66–68] and take up residence in the perivascular space of the CNS. In fact, limiting the bidirectional trafficking of HIV-infected macrophages into the CNS and restricting HIV replication within CNS compartments is of critical importance in reducing the production of viral proteins and limiting the CNS inflammation that drives HAND [64]. Of note, drug abuse exacerbates the pathological CNS changes found in patients with HAND [69]. For example, there is experimental and clinical evidence indicating that HIV neuropathogenesis and neurocognitive deficits are exacerbated with co-exposure to methamphetamine (meth) [63]. Furthermore, meth induces HIV transcription, potentiates HIV protein-mediated oxidative stress pathways in the brain, and increases proinflammatory cytokines, [65,70]. HIV-1, and the viral proteins Tat and gp120, also have complex and variable effects on the drug efflux proteins forming neurovascular unit cells: Tat induces P-glycoprotein (P-gp) and Multidrug resistance protein 1 (MRP-1) expression and functions in brain microvascular endothelial cells [71], although HIV can downregulate P-gp expression in primary human astrocytes [71]. How molecular alterations in the BBB disrupt the function of the cells forming the tight-junction barrier remains unknown, especially the extent to which HIV-1 proteins and meth-induced alterations in ART penetration result from disruptions to BBB integrity and/or drug transporter function is still uncertain.

Also, past work has shown that microglial activation may be related to BBB disruption [72]. For instance, Sumi et al. studied the effects of LPS on BBB functions in an *in vitro* co-culture system using rat brain microvascular endothelial cells (RBEC) and microglia. Treatment with LPS on the outside of the insert (abluminal side) in both the RBEC monolayer and RBEC/microglia co-culture had no effect on transendothelial electrical resistance (TEER) in the RBEC monolayer. Nevertheless, treating the RBEC/microglia co-culture in the same way caused TEER to decrease in proportion to the number of microglial cells present. Moreover, LPS had no effect on the permeability coefficient of sodium-fluorescein (Na-F) in the RBEC monolayer, while it increased Na-F permeability in the RBEC/microglia co-culture. Immunostaining for the tight junction proteins Zonula occludens-1, Claudin-5, and Occludin showed that these proteins were continuously distributed along the cell border in RBEC co-cultured with microglia, but when treated with LPS, this expression pattern was restored to a linear shape by adding diphenyleneiodonium chloride, a reduced nicotinamide adenine dinucleotide phosphate (NADPH) oxidase inhibitor. Given that tight junctions and efflux transporters are the main machinery underlying the function of the BBB [73], these results suggest that activated microglia probably produce ROS through NADPH oxidase, which impairs BBB function [72].

Microglia can also become primed towards reactive phenotypes in neurodegenerative diseases and cause neuronal damage, either *via* systemic factors or through neutrophils invading the injured BBB [24,49]. This can make it difficult to correlate specific microglia phenotypes with changes in the BBB. Furthermore, as already characterized in other neuronal diseases, phagocytic microglia may engulf endothelial cells and other neurovascular unit components [52]. Moreover, some work has analysed how the TNF-α released from activated microglia affects the BBB integrity in an *in vitro* co-culture system with the MBEC4 mouse brain capillary endothelial cell line. MBEC4 cell permeability to Na-F increased when microglial cells were activated by LPS, and this effect could be blocked by a neutralising antibody against TNF-α, thus indicating that TNF-α adds to BBB dysfunction [74]. In addition, the brain cells of neurodegenerative disease patients show decreased CD200 expression in association with microglial activation. Of note, CD200 is expressed on several cell types and its receptor, CD200R, is expressed on microglia and this interaction modulates inflammation and macrophage function. Moreover, Denieffe et al. observed an increase in TNF-α and IFNγ expression in CD200-deficient mice. Given that these molecules promote classical microglial activation and IFNγ is not normally produced by cells resident in the brain, this increase was probably because of T cells and macrophages entering the CNS, facilitated by increased BBB permeability [75].

## 5. Mechanisms of HIV in neuronal damage

Direct HIV-mediated neurotoxicity is related to the interaction between neurons and viral proteins gp120, viral surface glycoprotein 41 (gp41), negative regulatory factor (Nef), Tat, Vpr, and Viral protein U (Vpu), resulting in neuronal injury or apoptosis and contributing to CNS pathology (Figure 1) [76]. During the process of HIV entry into host cells, the viral envelope proteins gp120 and gp41 may damage other neurons in close proximity to them. More of these damaging viral proteins are released when viral replication is high leading to the release of viral particles from these infected cells [77] through a direct mechanism involving the induction of ROS production and increased cell death [78–81]. Elevated levels of ROS increases DNA nucleic acid oxidation, causing DNA instability, and also inhibits DNA repair by eliminating DNA glycosylase 1 [82]. Gp120 and Tat further contribute to neurotoxicity by increasing lipid peroxidation, leading to the accumulation of ceramide [83]. Vpr protein provokes G_2_/M arrest and plays a role in the infection of macrophages [84], HIV transcription, and apoptosis [85,86]. Finally, Vpu induces virion release by preventing the action of host restriction factors [87,88], downregulating CD4 during the late stages of HIV-1 infection [89], and impeding Nuclear factor-kappa-light-chain-enhancer of activated B cells (NF-κB) activation [89,90].

Tat, gp120, and gp41 are also involved in increased production of inflammatory cytokines and chemokines in astrocytes and microglia which mediates indirect CNS pathology [91]. Gp120 induces TNF-α, IL-6, IL-8, and monocyte chemoattractant protein 1 (MCP-1; Figure 1) [92] and excites A-type transient outward K + currents resulting in cell death [93], both in a ROS-dependent fashion [94]. Interestingly, ROS levels inversely correlate with CD4^+^ cell counts [95–98] perhaps because of the accumulation of DNA damage in these cells resulting from the increased generation of ROS and elimination of DNA repair enzymes [82].

## 6. The involvement of reactive oxygen species and microglia in HIV-associated neurocognitive disorder

Many links have been described between HAND in AIDS patients and nitrosative stress or the overabundance of nitrosative species during neuroinflammation [70] caused by bother the direct and indirect mechanisms of HIV neuronal damage during apoptosis, as explained above. Recent studies have clearly connected the CNS pathology of HIV and the effects of reactive nitrogen species (RNS), specifically NO. Of note, glial cells express inducible NOS (iNOS), which produces high amounts of NO [99]. Furthermore, nitrosative stress in microglia and astrocytes can be promoted by viral proteins such as Tat, gp41, and gp120, which are involved in iNOS induction [95]. In a murine model, Mangino et al. recently proposed that extracellular Nef causes neuronal injury by upregulating iNOS expression and the production of NO [100–102]. Indeed, iNOS expression is linked to HAND, and an association between nitrosative stress and a neuroinflammatory environment in the brain of HIV-1-infected patients has been confirmed [103,104].

An important work has shown that NO blood levels were higher in HIV-1-infected children with a high viral load [105] which, together with other studies in AIDS patients, suggests that a role for NO in the death and functional deterioration of lymphocytes in these patients should be considerered [77,105]. Another research has demonstrated that NO may increase HIV-1 replication *in vitro* [104]. For example, the addition of NO donors and TNF-α to mitogen-activated HIV-1-infected human peripheral blood mononuclear cell (PBMC) cultures significantly increased viral replication, which was reversed by the addition of iNOS-specific inhibitors [104,106]. Thus, as with ROS, NO may also contribute to HIV-1 replication, especially in proinflammatory settings [104], while the effects of RNS on the brain and other organs of HIV-1 infected patients seems to depend on their concentration and the length of exposure.

The mechanisms of neuronal damage and neurotoxicity after HIV infection are also interesting topics. It appears that the NO radicals produced during the immune response to infection are involved downstream in the neuron damage caused by HIV infection [70]. Although the concentration of radical species is controlled by the antioxidant systems implicated in their elimination in basal conditions, HIV infection creates an imbalance resulting in the overproduction of ROS and RNS and underproduction of endogenous antioxidant defences such as glutathione [70]. This disequilibrium exposes neurons to high levels of oxidative species which can produce neurodegeneration, alterations in the composition of lipid membranes, and post-translational protein modifications [107]. Oxidative stress can also influence the progression of neuronal degeneration because activated microglia can produce and release ROS and RNS in reactions catalysed by nicotinamide adenine dinucleotide phosphate [108]. These highly reactive free radicals can cause neuronal cell death and are implicated in the pathogenesis of neurodegenerative diseases and HAND [108]. Thus, the CNS is vulnerable to the excess free radicals and ROS produced by activated microglia as part of the inflammatory response.

Numerous studies have shown how HIV infection generates pronounced oxidative stress in HIV-infected laboratory models seen as high levels of ROS production in monocytes and a strong increase in ROS levels in HIV-infected cell cultures [109]. Similarly, ROS and RNS levels are high in the CNS after HIV infection [110]. Reactive species can also act as signalling molecules that lead to the expression of factors that deregulate the inflammatory response. According to Staal et al., the most acute reduction in total antioxidant capacity was observed in subsets of CD4^+^ and CD8+ T-lymphocytes [111], and low CD4 T-cell counts correlated with high oxidative stress levels [96]. Thus, the redox balance is key in the activation, proliferation, and differentiation of T cells [112]. Increased 4-hydroxynonenal levels [107] and high levels of oxidative stress markers were also detected in CSF and brain frontal cortex samples collected during the autopsy of HIV-infected individuals [82]. Interestingly, similar effects were observed in the NL4-3Δ transgenic rat model which expresses a HIV proteome devoid of the Gag-Pol polypeptide. Kline et al. discovered elevated levels of superoxide anions in the aortas of these animals by using electron spin resonance spectroscopy with a 1-hydroxy-3-methoxycarbonyl-2,2,5,5-tetramethylpyrrolidine probe [83] and fluorescent microscopy with dihydroethidium dye in the lungs [113]. Taken together, this work indicates that HIV-1 actively interferes (and plays and crucial function in) the development of a normal oxidative stress response. Moreover, the presence of increased ROS promotes the proliferation of astrocytes and microglia and induces proinflammatory activity cytokine secretion (including IFN-γ, TNF family members, and IL-1β, IL-6, IL-8, and IL-12) in the latter, leading to cell damage and progressive neurodegeneration [112].

Microglia and macrophages infected *in situ* produce gp120, Tat, and Vpr proteins which, as previously mentioned, can affect CNS [107,114] (Figure 1 and Table 2). The gp120 viral protein is a neurotoxic surface protein that can cause excitotoxicity which is strongly implicated in HAND. Excitotoxicity happens when excess glutamate activates neuronal N-methyl-D-aspartate (NMDA)-coupled ion channel receptors (NMDARs), resulting in unusually high calcium influx. This leads to further free radical NO formation, mitochondrial damage, and the generation of ROS concomitant with lipid peroxidation and caspase activation which produces neuronal damage [115] (Figure 2). Gp120 stimulates excitotoxicity by binding to NMDARs on neurons, resulting in excessive calcium flow, while Tat phosphorylates NMDARs which potentiates glutamate excitotoxicity [116]. Both Tat and Vpr have also been implicated in neuronal damage, with neuronal apoptosis being especially associated with Tat [117] and Vpr-induced oxidative stress which aggravates HIV-1-induced symptoms, thus affecting the pathogenesis and progression of HAND.

## 7. Role of inflammation in HIV-associated neurocognitive disorder

Inflammation is a common event observed in CNS cells in patients with HAND which is caused by innate immune system activation [118]. HIV-1 enters in CNS *via* the trafficking of infected monocytes and lymphocytes across the BBB [119] (Figure 1); in fact, the principal reservoir for the infection in the CNS is macrophages and microglia [120]. Parenchymal microglia are long-lived cells in the CNS, and current data indicates that perivascular microglia slowly turnover at the same rate as blood monocytes [121]. Williams et al. demonstrated that, in a primate model of simian immunodeficiency virus infection in which the infection was skewed towards perivascular macrophages [122], in HIV-1 infection immunopositivity was restricted to the perivascular compartment or parenchymal microglia [121,123]. It is important to highlight that infection of long-lived macrophages and microglia in the CNS promotes neurological dysfunction and serves as a long-term reservoir resulting in persistence of HIV-1 within these sanctuary sites. This is one of the main obstacles in the treatment and possible elimination of these infections [124].

Doubt remains about whether rounds of infection and viral amplification are due to the accumulation of HIV-1 cells in brain viral reservoirs or because of the continued influx of infected cells from the blood stream. Whereas monocyte-derived macrophages are most commonly used to model HIV-1 infection, HIV-1 replication has also been described in cultured primary microglia isolated from adult, infant, or foetal brains [125]. The presence of HIV-1 DNA has also been described in astrocytes and neurons isolated from HIV-1 encephalitis brain tissue by LCM [69,124]. However, it is not clear if this indicates a true latent infection or what role it might play in the pathogenesis of the infection. Accordingly, neurological dysfunction in HAND appears to be an indirect effect involving microglial infection and activation, inflammation, numerous molecular mechanisms, and multiple overlapping pathways.

Previous studies have shown that HIV-1 infection in the CNS induces the release of the proinflammatory cytokines IL-1β and IL-8 [124]. Furthermore, it has been well documented that IL-1β is released into the supernatants of glial cultures after exposure to HIV-1 glycoproteins [126]. Aside from its role in transcriptional signalling, HIV-1 Tat has been extensively implicated in the inflammation associated with HIV-1 [127,128]. Furthermore, Yang et al. have shown that exposure of monocytes to Tat promotes IL-1β release [129]. Despite the use of ARTs, Tat and other viral proteins remain in the brain and peripheral organs of HIV-1-infected patients, thus contributing to the latent inflammation correlated with HAND. Additionally, myeloid cells such as microglia are the principal target for HIV-1 infection in the CNS and these can release and take up soluble Tat [124].

As mentioned above, several pro-inflammatory cytokines such as IL-1β, TNF-α, IL-6, Granulocyte-macrophage colony-stimulating factor, and Macrophage colony-stimulating factor are increased in the CNS and/or CSF of HAND patients [121,130]. This increase may result from direct viral infection or by the action of shed viral proteins which stimulate uninfected mononuclear phagocytes to express elevated levels of cytokines [131]. Once this process commences, many of these cytokines produce autocrine or paracrine positive feedback to further increase their expression in macrophages/microglia, leading to a pro-inflammatory environment in the CNS. In addition, several of these have direct or indirect neurotoxic properties and may also contribute to inflammation processes and subsequent neuronal damage [121].

TNF-α is one of the most important proteins in the inflammation and neurotoxicity associated with HAND and its levels are elevated both in the brain tissues and CSF of HAND patients [132]. Moreover, TNF-α expression is increased by the action of gp120 and Tat in macrophages [133]. TNF-α causes injury to the BBB and induces expression of adhesion molecules on astrocytes and endothelial cells, thus causing HIV-1-infected macrophages to migrate into the CNS [134]. In addition, TNF-α up-regulates the expression and release of several chemokines in the CNS, including MCP-1, a chemoattractant for monocytes and macrophages [121]. Thus, by augmenting the BBB permeability and inducing adhesion molecule and chemokine expression, TNF‐α plays an important role in the entry of HIV‐infected cells into CNS [135].

TNF-α also has toxic effects on human neurons [136] by causing over-stimulation of glutamate receptors such as NMDArs, which are expressed on neurons. TNF-α also increases the release of the excitotoxic neurotransmitter, glutamate, from astrocytes and microglia [137]. Previous studies have shown that HIV-1 infected macrophages are the cause of extracellular glutamate and indeed, glutamate concentrations in the CSF of HIV-1 infected patients are higher compared to uninfected controls [138–141]. Generation of excess glutamate by HIV-infected macrophages in HAND may contribute to neuronal apoptosis and cell death. TNF-α also inhibits glutamate uptake by astrocytes, causing the extracellular levels of the latter to increase [139]. The numerous effects of TNF-α causes NMDAr over-production resulting in Ca^2+^ mobilisation and the formation of NO and ROS in neurons [136].

Finally, TNF-α can also induce adhesion, chemoattraction, and activation of other inflammatory cells, including macrophages, by regulating the expression of fractalkine ligand CX3CL1, which then binds its ligand, C-X3-C motif chemokine receptor 1. Furthermore, expression of the latter appears to be associated with lymphocytes and monocytes and this receptor plays a major role in the monocyte survival [142,143]. These data suggest that TNF-α is an important proinflammatory protein which plays crucial roles in macrophage stimulation and recruitment, acts as a direct neurotoxin, and which can lead to both astrocyte activation and decreased astrocyte uptake of the excitotoxic neurotransmitter, glutamate which, in turn, potentiates glutamate neurotoxicity [121].

## 8. Major research frontiers in HIV-associated neurocognitive disorders

### 8.1. Exosomes and microRNAs & LncRNAs

MicroRNAs (miRNAs) belong to the class of noncoding RNAs and are about 19–25 nucleotides in length. They are generated from endogenous primary miRNA precursors by RNA polymerase II when its acts on genomic DNA sequences. Primary miRNAs are catalysed and processed into single-stranded mature miRNAs by two ribonuclease III enzymes: Drosha and Dicer [144]. Interestingly, one of principal roles of miRNAs may be to exercise regulatory effects on virus propagation and replication, and they are important agents in gene silencing and in post-transcriptional protein regulation. During viral infection, some of the main viral genes involved in its pathogenesis also cause miRNA production by interacting with the virus or host cell mRNAs [145].

Mounting evidence suggests that these miRNAs may be useful biomarkers for screening and monitoring of HIV-infected patients during various infection phases based on the idea that disease conditions likely change the physiological expression patterns of these transcription-regulating molecules. Thus, dysregulated miRNA expression profiles could be used to distinguish early and late HIV infection in affected patients. Moreover, their resistance to degradation, tissue specificity, fast response to the cellular environment, and predictable effects on biological pathways also make miRNAs ideal candidates for monitoring processes and aberrations in biological systems [146]. Extracellular miRNA can be detected in all biofluids in association with lipoproteins, argonaute family proteins, or exosomes, which adds to their value as resistant molecules capable of tolerating common sample handling problems without degradation [147].

Recent studies have reported that patients on cART still present a large buildup of HIV-1 RNA within infected cells; this results in the production of defective viruses over time, which may contribute to HAND [148]. Interestingly, these viral noncoding RNAs may be exported from infected cells through extracellular vesicles (EVs), including exosomes [148]. Exosomes are a type of EV, measuring about 30–120 nm in diameter, which are found throughout various body fluids, such as CSF [149]. They are known to carry nucleic acids (RNA, miRNA, and DNA), functional proteins (including those of viral origin), and other cellular products. These vesicles form within multivesicular bodies in the late endosome and are released by almost all types of cells during normal cellular functioning and specifically, in response to cellular stressors.

Regarding the role of miRNAs in HAND, one study examined the impact of Tat upon the expression of selected miRNAs in primary cortical neurons *in vitro* [150]. Tat was found to upregulate mir-128a, which in turn inhibited expression of SNAP25, a presynaptic protein. Another study examined caudate and hippocampus brain tissue from rhesus macaques with (*n* = 4) and without (*n* = 4) simian immunodeficiency virus encephalitis (SIVE), and caudate tissue from human HIV-negative controls (*n* = 6) or patients with both HAND and human immunodeficiency virus encephalitis (HIVE; *n* = 5), although only 3 patient samples were used for the microarray analysis [151]. While three miRNAs were found to be elevated in SIVE and HIVE (miR-142-5p, miR-142-3p, and miR-21), the study primarily focussed on miR-21 (an miRNA which is largely known for its link to oncogenesis), which was significantly upregulated in both HIVE and SIVE samples and was specifically found in neurons. Further analysis revealed that it also stimulated N-methyl-D-aspartic acid receptors, leading to subsequent electrophysiological abnormalities. The authors showed that miR-21 targets the mRNA of myocyte enhancer factor 2 C (MEF2C), a transcription factor which is crucial for neuronal function, ultimately reducing the expression of this mRNA—a finding also supported by immunohistochemistry findings in neurons from HIVE and SIVE brains. Similarly, Noorbakhsh et al. identified the differential expression of multiple miRNAs in frontal lobe white matter by comparing HIV-negative and HIVE cases matched by age and sex [152], in an expression profiling study using the standard two-fold cut-off as the threshold for further analysis.

In terms of EVs, other work has reported that exosome-associated HIV-1 Nef provoked TNF-α production in recipient PBMCs and elicited bystander apoptosis in recipient CD4^+^ T-cells [153]. Furthermore, exosomes originating from infected cells contain TAR RNA, which shuts down the PKR/eIF2α pathway, and activates TLR-3 and the NF-κB pathway in uninfected recipient cells [154]. Additionally, exosomes from HIV-1-infected cells disrupted the BBB in an *in vitro* model [155] and a 2014 study also showed that exosomes from HIV-1-infected cells promoted HIV-1 replication in recipient cells [156]. HIV-1-infected dendritic cells can mediate viral trans-infection *via* fibronectin and galectin-3 [157] and exosomes from both uninfected and infected cells have been shown to activate latent HIV-1 in infected cells [157]. Thus, ongoing work is looking at which RNAs and proteins from exosomes could be associated with HIV-1, and their functional effects in recipient cells. A recent study has shown that exosomes from infected T-cells were enriched in histones and contained numerous cyclin dependent kinases and Src family kinases that were absent from uninfected cell exosomes [157]. Several long noncoding RNAs were also observed in exosomes from infected T-cells that were not present in monocytes, while mitochondrial DNA has been found in large EVs from both uninfected and infected cells [157].

MiRNAs regulate gene expression at the post-transcriptional level by binding to the 3′-UTR and/or the coding regions of their target mRNAs, and hundreds of miRNAs have been found in exosomes [158]. Importantly, exosomal miRNAs can repress mRNAs in target cells and subsequently influence target cell function and have also been implicated in several cellular processes and human diseases, including cell migration, differentiation, viability, aging, neurodegeneration, and immune disorders [158]. Moreover, recent work has demonstrated that EV-miR-9-induced downregulation of phosphatidylinositol-3,4,5-trisphosphate 3-phosphatase (PTEN) expression enhanced microglial migration [159]. Given the ability of EVs to spread miRs and toxic proteins within the CNS, EV-miRNA-mediated microglial impairment could be envisioned as one of the many contributors mediating accelerated neurodegeneration in HAND. Of note, this study identified a unique role for miR-9 in mediating microglial migration *via* EVs released from Tat-stimulated astrocytes [159]. Astrocytes exposed to HIV Tat induce the expression and secretion of miR-9 in EVs, which are then taken up by the microglia and, in turn, lead to increased microglial migration in the brain [159]. MiR-9-mediated regulation of microglial migration suppressed the target protein, PTEN—a critical suppressor of cell motility. These results not only explain the mechanisms underlying the roles of EV-miRNAs in microglial dysfunction, but also sets the stage for future testing of EV-based therapeutic strategies using anti-miRNA or siRNA oligos for treating HAND.

### 8.2. Pharmacological strategies to target infected microglial cells

**Figure 3.**
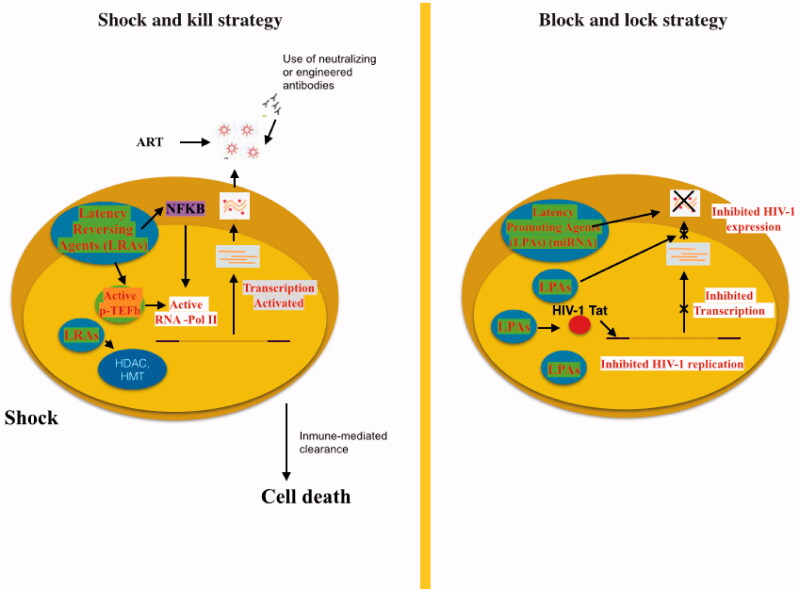
“Shock and kill” strategy involves activating viral replication to eliminate reservoirs and to target latently-infected cells. LRAs have an important role in the reactivate transcription for the “shock”. LRAs promote chromatin decompaction and ARN pol II recruitment to induce virus transcription. ART is maintained during this phase to clear reservoir without virus propagation in other cells. The “kill” can be enhanced by stimulation of the cell-mediated immune response or by using neutralising and/or engineered antibodies. The “block and lock” strategy relates on the induction of a state of deep-latency to prevent HIV-1 transcription. LPA inhibit various step of virus replication, transcription by Tat inhibition and RNA export. One promising LPA is a miRNA which inhibit virus expression.

Targeting all the HIV-1 reservoirs, such as microglial cells in the brain, is crucial to achieve either a sterilising or a functional cure, because these cells are potential sources of HIV-1 reseeding in the blood. In addition, production of the virus in these cells has been associated with HIV-1 resistance and the development of HAND. A main concern is to prevent deleterious neuroinflammation associated with infected microglial cells [160]: to date, two plans have been suggested for this purpose, the “shock and kill” and “block and lock” strategies (Figure 3).

#### 8.2.1. The shock and kill strategy

The shock and kill strategy is a new therapeutic approach based on the idea that the latent HIV provirus could be switched into an active form (shock) which would be susceptible to eradication (kill) through the humoral immune response, CD8+ T cell-mediated lysis, virus-induced apoptosis, and activation-induced cell death [161]. To this end, different latency-reversing agents (LRAs) or “shock” inducers have been suggested [162] including histone deacetylase (HDAC), histone methyltransferase (HMT), and DNA methyltransferase (DNMT) inhibitors. HDAC enzymes are responsible of removing acetyl groups from histones, which favours the formation of a compacted, transcriptionally repressed chromatin structure [162]. HDAC inhibitors, including suberanilohydroxamic acid (SAHA) or DNMT inhibitors could be used alone to reactivate HIV gene expression alongside efficient cART [162]. For instance, co-treatment with SAHA and the global T cell activator 12-O-tetradecanoylphorbol-13-acetate (TPA) synergistically purged HIV-1 proviruses in latently HIV-1-infected cells [162].

Other activators of NF-kB such as prostratin have been used in combination with HDAC inhibitors to reactivate HIV but were unable to provoke immune activation [163]. However, the concomitant use of protein kinase C agonists (prostratin, bryostatin-1, and ingenol-B), which are known to activate the NF-κB signalling pathway and Positive transcription elongation factor B (P-TEFb), utilized alone or in combination with P-TEFb-releasing agents (HMBA and bromodomain and extra-terminal [BET] inhibitors JQ1, I-BET, and I-BET151), leads to synergistic HIV reactivation from latency [163]. Sequential treatment with the DNMT inhibitor 5-aza-2′-deoxycytidine and HDAC inhibitors reactivates HIV-1 also had the same effect [164]. Similarly, HIV-1 recovery in quiescent CD4^+^ T cells during highly active ART-treated patients with an undetectable viral load using chaetocin and BIX-01294 (specific inhibitors of HMT Suv39H1 and G9a, respectively) has also been shown [165].

TNF-α-based therapies could be another therapeutic approach whereby the combination of HDAC inhibitors or HMT inhibitors with TNF-α would interrupt HIV-1 latency by generating the activation of transcriptional activators like NF-κB, thus preventing the formation of heterochromatin and increasing HIV-1 long terminal repeat transcription, in turn leading to viral purge [166].

Strategies which focus on several cellular proteins implicated in the epigenetic control of viral gene expression usually result in the amplification of HIV-1 reactivation. Epigenetic mechanisms such as histone post translational modifications (e.g. acetylation and methylation) and DNA methylation of the proviral DNA are involved in the establishment of HIV-1 latency. The better understanding of epigenetic mechanisms modulating HIV-1 latency could give clues for the complete eradication of these latent reservoirs. Nonetheless, there are several obstacles to this tactic. For instance, it may only reactivate only a small subset of the latent HIV genome for possible elimination. Regardless, this approach could still be used in parallel to other procedures to achieve a fully effective cure [166].

An important limitation of the use of the shock and kill strategy is that it reactivates both microglial cells (leading to neuroinflammation because of the secretion of pro-inflammatory factors) and some viral proteins such as Tat and gp120, which are neurotoxic. One way to prevent this inflammation would be to improve cART by targeting HIV-1 transcription or by inhibiting RNA export to counteract the effects of these pro-inflammatory cytokines and to prevent synthesis of viral proteins [167].

#### 8.2.2. Block and lock strategy

In contrast to LRA, the “block and lock” strategy is as a novel approach to functionally cure HIV in which chemical agents “block” the ongoing viremia during cART by “locking” the HIV promoter in a super latency state that is resistant to reactivation stimuli. Thus, didehydro-cortistatin A (dCA) has been studied a specific and powerful Tat inhibitor [168] which specifically binds the TAR-binding domain [169] to reduce the residual levels of HIV transcription, establishing a permanent state of latency and delaying viral rebound after cART interruption in HIV + humanized BLT mice [170]. This could be advantageous in situations of therapy non-compliance or short periods of discontinuation because it can restrict continual replenishment of the CD4^+^ T cell reservoir [171]; dCA would restrict the increased latent viral reservoir longevity and persistence promoted by cART-treatment by blocking new CD4^+^ T cell infections [171]. Importantly, Tat has no cellular homolog and so, using dCA to “block and lock” HIV should not silence other regulatory pathways crucial to fighting other infections. Furthermore, Akt activation favours HIV-1 reactivation in quiescent CD4^+^ T cells, monocytes/macrophages, and microglia, i.e. the principal HIV-1 cellular reservoirs [172]. Thus, blocking Akt activation in HIV-1 infected cells with Akt and HIV protease inhibitors [172] (favoring the lock stage) will reduce cell viability, making it possible to eliminate these infected cells. Thus, the block and lock approach may be a useful addition to current therapeutic management strategies.

### 8.3. Calcium dysregulation and mitochondrial dysfunction

Ca^2+^ is a crucial signalling messenger which plays a critical role in the bioenergetics of neuronal function. Increased intracellular Ca^2+^ always precedes changes in oxygen consumption rates in stimulated neurons [173], while transferring Ca^2+^ from the endoplasmic reticulum (ER) to mitochondria stimulates oxidative metabolism and metabolically energizes mitochondria. Thus, the mitochondrial-associated ER membrane plays an important role in the maintenance of cellular homeostasis by regulating Ca^2+^ transfer and energy metabolism [173]. HIV-1 proteins, including Tat and gp120, cause Ca^2+^ dysregulation, damage, and death in both neurons and glial cells, as indicated by abnormal and disproportionate Ca^2+^ influx and increased intracellular Ca^2+^ release [174]. In rat hippocampal neurons, Tat alters intracellular Ca^2+^ levels by triggering its release from the ER *via* the inositol-1,4,5-trisphosphate receptor (IP3R) [174,175], which is subsequently then taken up by the mitochondria, resulting in mitochondrial Ca^2+^ overload, the accumulation of ROS, and oxidative stress. Furthermore, Tat-induced neuronal apoptosis is mediated by dysregulated Ca^2+^ signaling [175] and in mouse striatal neuron cultures, Tat increased intracellular Ca^2+^ and induced instability in the mitochondrial inner membrane potential, leading to synaptic damage [175].

Also, another important study showed that the activation of HIV-1 beta-chemokine co-receptors stimulates signalling processes in human foetal microglia through a Ca^2+^-dependent mechanism; beta-chemokines, RANTES (regulated upon activation, normal T cell expressed and presumably secreted), and eotaxin activated a nimodipine sensitive Ca^2+^ influx pathway in human foetal microglia. HIV-1 Tat protein mimicked chemokine-mediated Ca^2+^ signalling and may modulate the migratory and secretory responses of microglia [176]. Ca^2+^ signalling dysregulation by viral proteins may also mediate upstream functional mitochondrial disruptions which could subsequently result in harmful hypermetabolic conditions. Of note, the mitochondrial abnormalities found both *in vivo* and *in vitro* were comparable with those associated with HIV infection [177,178]. Recent work has shown significant changes in the synaptic mitochondria isolated from HIV-1 transgenic rats, including abnormalities in the expression of ETC-complex subunits [178]. In addition, increases in the protein expression of tricarboxylic acid cycle (TCA) and fatty-acid metabolic processes were also noted and tie in with other findings that HIV-1 Tg rats had higher oxygen consumption rates than their littermate controls [178].

Different changes in mitochondrial morphology are associated with CNS HIV infection [179]. For example, mitochondrial size was higher in the frontal cortex of HAND patients, suggesting that mitochondrial fusion was preferred over fission in these individuals. This hypothesis was supported by the fact that mitochondrial fission proteins such as Dynamin-1 like were decreased and mitochondrial fusion protein like Mitofusin 1 were increased in these individuals. Notably, these alterations were specifically identified in neuronal mitochondria [179] and mitochondrial hyperfusion protects cells and supports mitochondrial ATP synthesis in response to various stresses [180]. Indeed, several lines of research seem to indicate that a loss and/or gain of function in mitochondrial biology contributes to HAND. For instance, mitochondrial function is often disrupted when ROS generation is increased during HIV neuropathogenesis, with increased oxidative and nitrosative stress having been shown early in HIV infection and throughout the progression of HAND [70]. These findings highlight the complexity of bioenergetics in the brain over the course of HIV infection as well as the fact that diverse brain regions metabolically respond in different ways.

Several lines of evidence have also implicated the role of ROS and mitochondrial danger-associated molecular patterns (such as the mitochondrial DNA released from damaged mitochondria), in activating inflammatory pathways such as NFKB1 and the NLR family pyrin domain containing 3 (NLRP3) inflammasome [181,182], that underlie neurodegeneration. One group reported that exposure to HIV-1 Tat mediates the priming and activation of the NLRP3 inflammasome in microglial cells and that blocking this pathway resulted in the abrogation of IL1B secretion [123]. Other results have shown gp120-induced NLRP3-dependent pyroptosis and IL-1β production in microglia during HIV infection. Indeed, inhibition of microglial NLRP3 inflammasome activation alleviated gp120-mediated neuroinflammatory factor release and neuronal injury [183]. Importantly, this work showed that chronic administration of MCC950 (a novel selective NLRP3 inhibitor) to gp120-transgenic mice not only attenuated neuroinflammation and neuronal death, but also promoted neuronal regeneration and restored their impaired neurocognitive function [183]. Thus, these data revealed that the NLRP3 inflammasome is important for gp120-induced neuroinflammation and neuropathology and suggest that NLRP3 is a potential novel target for the treatment of HAND.

Signalling of the GU-rich single-stranded RNA from the HIV long terminal repeat region (ssRNA40) through Toll-like receptors 7 and 8 has been shown to induce the secretion of IL-1β in primary monocytes [184]. Studies that examined the activation of microglial cells by HIV ssRNA40, and the potential subsequent neurotoxicity found that exposure of human primary microglia to ssRNA40 activated the NLRP3 inflammasome [184]. The expression and extracellular secretion of the pro-inflammatory cytokines IL-1β and Interleukin-18 and neurotoxic cytokines TNF-α, IL-1α, and C1q increased following exposure to ssRNA40, and exposure of these cytokines (secreted from ssRNA40 culture supernatants) to human primary microglia induced toxicity. Moreover, microglial inflammasome activation increased ROS generation and led to a loss of mitochondrial membrane potential and mitochondrial integrity. Treatment with ssRNA40 blocked autophagy/mitophagy-mediated negative regulation of the NLRP3 inflammasome and caused the release of inflammatory cytokines, caspase-1 activation, and pyroptotic microglial cell death. Thus, HIV ssRNA-mediated activation of microglial cells can contribute to neurotoxicity and neurodegeneration *via* the secretion of inflammatory and neurotoxic cytokines [184].

The molecular mechanism(s) underlying the impairment of mitophagosome-lysosome fusion and poor clearance of mitophagosomes is still poorly understood. However, this process is likely the result of the accumulation of HIV-1 Tat in infected macrophages and microglia that transport and secret it across the BBB from the systemic circulation [185].

A previous work indicate HIV-1 Tat enters neurons *via* receptor-mediated endocytosis. Endocytosis is a very rapid and early event, which results in the release of HIV-1 Tat into the cytoplasm and uptaken into the nucleus, most probably through the mechanisms involving the high H^+^ gradient maintained by vacuolar H^+^-ATPase [185]

Recent results have established a novel link between mitochondrial dysfunction, defective mitophagy, and microglial activation, thereby suggesting that interventions aimed at blocking mitochondrial dynamics and/or increasing the clearance of damaged mitochondria may be promising therapeutic targets for abrogating HIV-1-mediated neuroinflammation and HAND progression [182]. Other work in primary human neurons has shown that HIV gp120 and Tat alter mitochondrial dynamics, resulting in incomplete mitophagy and failure to eliminate damaged mitochondria. Neurons have a high energetic demand that depends heavily on mitochondrial function [186], and mitochondrial damage is associated with a loss of mitochondrial membrane potential [187]. Neurons incubated with HIV gp120 and Tat exhibit a significant reduction in TMRE-positive cells, indicative of parkin-targeted mitochondria with a decreased Δψm [187]. There is also compelling evidence that impaired mitophagy and microglial activation plays a central role in normal aging and the development of neurodegenerative disorders [187]. This research must continue to help unravel the molecular basis of the association between mitophagy and HIV in order to design novel approaches to counteract the accelerated aging and neurocognitive disorders associated with HIV infection of the brain [184].

### 8.4. Drugs of abuse

There is evidence that recreational drug use has a synergetic effect on the progression and severity of HAND [188]. Like HIV, drugs of abuse target the CNS and alter both neuronal and glial function. Recent clinical work has demonstrated that HIV patients who abuse drugs have more rapidly progressing disease, with higher viral loads, and increased cognitive damage [176,189]. In the simian immunodeficiency virus (SIV)-macaque model, exposure to opioids and meth raised brain and CSF viral loads [189]. Cocaine, meth, and opioids can raise HIV replication rates in rodent models and *in vitro* studies [190] which supports the idea that drug abuse may increase neuroinflammation and neurodegeneration, probably also disrupting brain metabolism. Independently, it has been also been shown that these drugs alter brain metabolism both during their active use and in periods of abstinence [191]. Moreover, alterations in brain metabolism have been detected in meth abusers [192], while cocaine reduces regional brain glucose uptake and glucose metabolism both in users and in mouse models of chronic cocaine use [190]. Furthermore, downregulation of mitochondrial oxidative phosphorylation genes was observed in the hippocampus of long-term cocaine abusers [193].

It has been suggested that energy pathways are impaired in drug users and that this problem may be more exacerbated in HIV patients who abuse drugs because of their already hypometabolic status. In mice, cocaine induces a metabolic switch from glycolysis to fatty acid oxidation and ketone metabolism, leading to enhanced activation of the TCA cycle, oxidative respiration, and a drastic increase in the consumption of ATP and acetyl-CoA in the nucleus accumbens [194]. Furthermore, work using an immortalized microglial cell line, showed that cocaine exposure in combination with HIV infection or exposure to HIV gp120 protein had an additive effect on cell energetics by raising ATP concentrations, glycolysis, and oxidative phosphorylation [194], and these increased energy requirements caused some microglia to transition to an activated phenotype. In rat hippocampal neurons, cocaine enhanced the Tat-induced decrease in mitochondrial membrane potential and exacerbated ROS production [195]. Conversely, exposure to Tat and cocaine increased mitochondrial metabolism in astrocytes, thus increasing ATP levels [196]. In the case of meth, a significant decrease in ATP was noted in rat cerebrocortical neuronal cells which was exacerbated in the presence of gp120 [186]. Astrocytes respond to meth by increasing mitochondrial fusion, resulting in enhanced oxidative capacity and increased ATP levels [197]. Finally, striatal neuronal cultures treated with morphine and Tat interacted to enhance mitochondrial dysfunction and increase intracellular Ca^2+^ [175,196], thus suggesting that drugs of abuse and HIV interact to disturb brain bioenergetics.

### 8.5. Combination antiretroviral therapy neurotoxicity

ARTs act upon key steps in the viral replication cycle, including viral entry inhibitors, nucleoside reverse transcriptase inhibitors (NRTIs), non-nucleoside reverse transcriptase inhibitors (NNRTIs), protease inhibitors, fusion inhibitors, CCR5 blockers, and integrase strand-transfer inhibitors. These drugs are combined into cARTs to help prevent the emergence of drug-resistant HIV mutant strains, although there is evidence that long-term use of some of these compounds can be neurotoxic, with patients reporting an unexpected improvement in cognition after interrupting cART [198]. Furthermore, even though the CSF suppression of viral replication in patients on a high efficiency CNS penetrance-effectiveness cART regime was higher, their neurological testing performance was inferior [199], implying that it may have had neurotoxic effects resulting in cognitive impairments. A magnetic resonance spectroscopy study showed that patients with HIV-1 who were taking NRTIs had lower levels of N-acetyl aspartate in the frontal white matter compared to HIV negative individuals and HIV-1 patients on a different cART regime [200]. In addition, prolonged periods of NRTI treatment were also related to diminished N-acetyl aspartate in these individuals [200]. Two animal models have been used to try to understand potential cART neurotoxicity: (1) SIV-infected pigtail macaques, either receiving early cART or no cART and (2) adult rats administered various combinations of antiretroviral (ARV) drugs [201]. These studies revealed that cART alone causes synaptic damage [201].

*In vitro* studies have also reported ARV toxicity, although their neurotoxicity varies depending on the individual drug and its drug class. For example, in mixed rat neuronal-glial cerebrocortical cultures, exposure to individual (or combinations of) ARV compounds reduced Synaptophysin and MAP2 expression, suggesting that they caused damage to dendrites and presynaptic terminals [186]. This neuronal damage manifested as beading, simplification of the dendritic processes, and neuronal shrinkage [201]. Additionally, various ARV drugs cause the accumulation of ROS in neurons, astrocytes, and oligodendrocyte precursor cells [200]. Similarly, various ARVs modify mitochondrial membrane potential, morphology, energy profiles, and toxicity [197]. Moreover, neuronal ATP was significantly diminished after exposure to ARV compounds [186] and exposure to various ARV drugs disrupts the mitochondrial function of presynaptic striatal nerve terminals by reducing maximal mitochondrial respiration and reducing ATP production [202]. In another study, exposure of human astrocytes to a combination of ARV compounds increased glucose utilisation, glycolysis, and mitochondrial metabolism, suggesting that their energy status had been enhanced during the treatment [201]. Unsurprisingly, in addition to the mitochondrial dysfunction associated with ARV compounds, they also induced ER stress [201]. As efforts to increase the efficacy of CNS penetrance by ARVs gains more attention, it is important to further explore the impact these compounds on brain metabolism and cognition.

Many patients using the anti-HIV drug efavirenz (EFV) NNRTI experienced impaired concentration and cognitive deficits [203]. It has been studied, the actions of EFV in neurons and glial cells and the possible interference with mitochondria, and the effect on glycolysis. EFV causes mitochondrial alterations in neuronal cell lines and primary neuron cultures, including decreased ATP production, and mitochondrial fragmentation and depolarization [203]. Another group found that it decreased mitochondrial membrane potential, reduced mitochondrial respiration, and raised ROS generation in both neuronal, astrocyte and microglia cultures [202]. Interestingly, EFV diminished neuronal ATP but increased astrocyte ATP levels by activating AMP-activated protein kinase, thereby upregulating glycolysis in astrocytes [202]. Another important study researched about the effects of teriflunomide (Teri) and monomethylfumarate (MMF) on monocyte/microglial activation and neurotoxicity. It demonstrated that Teri and MMF reduced the secretion of chemotactic and pro-inflammatory cytokines in a co-culture system of microglia with HIV-transduced monocytoid cells as demonstrated by the decreased neurotoxicity of this supernatant in human foetal neurons [204]. The effects of these drugs on cytokine levels may have implications for subsequent recruitment of inflammatory cells to the CNS and the aggravation of neurodegeneration [205].

### 8.6. Autophagy

The cellular autophagy pathway can be modulated for HIV-1 therapy and vaccine development, although this task is complex because of many interactions involved [206]. Nevertheless, studies have revealed that modulation and cautious monitoring of the autophagy pathway could become an alternative to cART in preventing HIV-1 associated neurological disorders [207]. Importantly, during the initial phase of HIV-1 infection, activation of autophagy could be disadvantageous to patients and advantageous (pro-viral) in many cell types. In later phases, HIV-1 inhibition of autophagy promotes the biogenesis of exosomes containing viral products, thus priming the infection of new cells and causing CD4^+^ T-cell cell death [208]. However, once HIV-1 becomes latent, autophagic induction might complement cART by activating quiescent viral reservoirs and preventing the export of HIV-1 products in exosomes [209]. Given that autophagy is differentially modulated by HIV-1 infection in different CNS cell types, a balanced approach to HIV-1 treatments that modulate autophagy is required. Targeting specific cell types in the brain in order to modulate autophagy could further enhance the effectiveness of this approach.

Some studies have found evidence for the accumulation of autophagosomes in the brains of mouse models and post-mortem HIV-1 patients, suggesting that increased autophagy may be associated with a autophagosome-lysosome fusion deficiency [210]. Furthermore, increased autophagy also seems to lead to neurite degeneration, while its restriction may cause a reduction in the number of autophagosomes, thus protecting neurites against degeneration [211]. However, although autophagy protects cells from stress-related toxicity, its over-induction can lead to cell death meaning that a delicate balance likely exists in which autophagy protects neurons from external toxins but its over induction can lead to cell destruction. This correlates with work showing that autophagy was impaired in primate brains with SIVE and post-mortem brain tissues obtained from HIV-infected patients with HIV-associated dementia [212]. Nevertheless, exposure of SK-N-SH cells to microglial supernatant resulted in the induction of autophagy [213], and exposure of these cells to virus-derived gp120 also resulted in increased autophagy. Moreover, increased levels of autophagic markers have been observed in brain samples from patients with documented HIV encephalitis and/or HIV-associated dementia. These data are consistent with the *in vitro* findings of Alirezaei et al. [213] and Espert et al. [214], who showed that both the autophagic proteins Atg-7 and Beclin-1 were increased in gp120-treated CD4^+^ T cells.

An increase in neuronal autophagy, or ineffective autophagy with the accumulation of autophagic products, was seen in the brains of patients with HIV-1-associated encephalitis compared to HIV-infected patients without encephalitis or non-HIV-infected controls postmortem [215]. These findings suggest a model for the development or progression of HIV-related CNS damage in which HIV-1 downregulates autophagy to facilitate viral replication during the permissive infection of susceptible cells; in contrast, different products of infection and cells that bind HIV-1 gp120 and that are nonpermissive to HIV-1 replication improve autophagy to eliminate toxic stress and maintain cell survival [215]. Thus, based on these data, an increase in autophagic activity leads to the development of HIV encephalitis and cognitive damage even though autophagy may help to sustain neuronal survival. This means that, when combined with antiretrovirals, drugs that reduce autophagic activity to homeostatic levels may help prevent or reverse the CNS deficits associated with HIV-1 infection. In summary, the dysregulation of autophagy during HIV infection is crucial in the pathogenesis of neuroAIDS.

## 9. Conclusions

Recent studies in AIDS patients indicate that microglial activation in the brain, with the resulting release of cytokines and chemokines, contributes to neurodegeneration and cognitive impairment. Since HIV is usually present in the brain because of the migration of infected monocytes and lymphocytes across the BBB, we should direct our efforts towards developing novel therapies designed to protect the integrity of the BBB during systemic HIV infection in order to help control infection of the CNS. Approaching the persistence of neurocognitive problems in patients infected with HIV is a major challenge. However, as ARTs improve, maintaining viral latency in the CNS to help protect neurons and reduce HAND is becoming a promising route towards improving the quality of life of these patients. One possible explanation for the pathogenicity of HAND is the presence of a CNS HIV reservoir, settled in the early stages of infection, with the resulting HIV-related brain damage leading to permanent CNS damage before ART commences. Thus, future prospective studies should try to diagnose and treat HIV earlier, aiming to optimize antiretroviral therapies in order to reduce residual viral infection and CNS neurotoxicity.

Exosomes have been studied in this field because of their capacity to carry an assortment of protein and RNA cargos and for their pivotal role in cellular signalling and regulation. Recent studies have shown that the biological cargo (primarily membrane proteins and microRNAs) of exosomes influences the pathogenesis of HIV infection. There is limited evidence that the exosomal budding and trafficking machinery is usurped by HIV during infection, although this mechanism cannot be ruled out [144]. Work has been carried out to inhibit HIV transcription by blocking exosomal packaging and transmission of viral elements; such inhibitors are likely to have a strong long-term positive impact on the health and quality of life of people living with HIV.

HIV does not directly damage neurons by productive infection, but rather, it acts by activating the release of inflammatory factors and ROS from infected macrophages and microglia. Even though a lot of recent work has tried to explain the different molecular mechanisms involved in varying neurodegenerative diseases, it is still unclear how excitotoxicity processes are implicated in viral infection during neuronal damage. The development of new therapeutic strategies to target redox markers will be crucially important in understanding whether the ROS present are the cause or the effect of the increased susceptibility of brain cells to infection in neurodegenerative disorders. Individual HIV-1 proteins released from infected cells in the CNS (such as gp120 and Tat) may play a leading role in HIV-related neurodegeneration. Of note for investigation in possible future studies, infected cells that do not effectively assemble infectious virions (because of ART) may still produce HIV-1 toxic proteins. However, this hypothetical scheme of HIV replication-independent production of HIV-1 viral protein is not yet well understood.

Several lines of evidence indicate that like HIV-1 (or its component proteins) and the use of meth, some ARVs used in cART can also induce oxidative and ER stress, compromise neuronal energy homeostasis, and trigger structural neuronal damage. In addition, the neurotoxic effects of individual viral proteins have been demonstrated in several publications. Many of these studies focussed on the viral proteins involved in diverse experimental systems, and as such, have shown that gp120, Vpr, Tat, and Nef can evoke neurotoxic effects to subsequently trigger numerous molecular pathways, likely contributing to neurodegeneration. Thus, the possible interaction of multiple HIV-1 proteins in this context would dramatically increase the complexity of the pathogenic cascades implicated in this pathogenesis. At the cellular level, neuronal synaptic regions can also be attacked by the excitotoxicity caused by the direct stimulation of neurons, reactive microglia, and astrocytes, therefore contributing to the coordinated processes of synaptic degeneration. Direct and indirect mechanisms of toxicity add to the damage in some neuronal sub-populations, resulting in neurocognitive deterioration.

An improved knowledge of the molecular mechanisms and different pathogenic processes underlying neuronal injury, neuroinflammation, excitotoxicity, and dysfunction during the progression of neuro-AIDS may lead to more specific and efficient treatment therapies, possibly even resulting in recovery. Indeed, many of the multiple sources of ROS that are activated by HIV may be regulated in similar ways, and hence, would also be susceptible to similar inhibition or stimulation treatments. Indeed, the status of antioxidant defense systems and impact of HIV-induced oxidative stress on the susceptibility to other viral infections has not yet been properly addressed. Thus, it is important to continue these studies because they could contribute to the future development of efficient antiretroviral treatments.

## Abbreviations

AIDS: acquired immune deficiency syndrome
ANI: asymptomatic neurocognitive impairments
ART: antiretroviral therapies
ARV: antiretroviral
ATP: adenosine triphosphate
BBB: blood brain barrier
BET: bromodomain and extra-terminal
Ca^2+^: calcium
cART: combined anti-retroviral therapy
CCR5: CC-chemokine receptor 5
CNS: central nervous system
CSF: cerebrospinal fluid
CXCR4: C-X-C-chemokine receptor 4
dCA: didehydro-cortistatin A
DCE-P: dynamic contrast enhanced perfusion
DNA: deoxyribonucleic acid
DNMT: DNA methyltransferase
EFV: Efavirenz
ER: endoplasmic reticulum
EV: extracellular vesicle
gp120: viral surface glycoprotein 120
gp41: Glycoprotein 41
HAD: HIV-associated dementia
HAND: HIV-associated neurocognitive disorder
HDAC: histone deacetylase
HIV-1: human immunodeficiency virus 1
HIVE: human immunodeficiency virus encephalitis
HMT: histone methyltransferase
IFN γ: Interferon γ
IFN-γ: interferon gamma
IL-1: interleukin-1
IL-1β: interleukin 1β
IL-6: interleukin-6
IL-8: Interleukin-8
iNOS: inducible nitric oxide species
LCM: laser capture microdissection
LPS: lipopolysaccharide endotoxin
LRA: latency-reversing agent
MCP-1: Monocyte chemoattractant protein type 1
MDMs: monocyte-derived macrophages
MEF2C: myocyte enhancer factor 2C
miRNA: MicroRNA
(MMF): monomethylfumarate
MMP2: matrix metallopeptidase 2
MMP9: matrix metallopeptidase 9
MND: mild neurocognitive disorders
MRP-1: Multidrug resistance protein 1
NADPH: reduced nicotinamide adenine dinucleotide phosphate
Na-F: sodium-fluorescein
Nef: negative regulatory factor
NF-κB: Nuclear factor kappa-light-chain-enhancer of activated B cells
NLRP3: NLR family pyrin domain containing 3
NMDA: N-methyl-D-aspartate glutamate
NMDAR: N-methyl-D-aspartate glutamate receptor
NNRTI: non-nucleoside reverse transcriptase inhibitors
NO: nitric oxide
NRTI: nucleoside reverse transcriptase inhibitors
PBMC: peripheral blood mononuclear cell
PCR: polymerase chain reaction
P-gp: P-glycoprotein
P-TEFb: Positive transcription elongation factor B
PTEN: Phosphatidylinositol-3,4,5-trisphosphate 3-phosphatase
QAlb: albumin quotient
RBEC: rat brain microvascular endothelial cells
RNA: ribonucleic acid
RNS: reactive nitrogen species
ROS: reactive oxygen species
SAHA: suberanilohydroxamic acid
SIV: simian immunodeficiency virus
SIVE: simian immunodeficiency virus encephalitis
START: strategic timing of antiretroviral therapy [trial]
Tat: trans-activator of transcription
TCA: tricarboxylic acid cycle
TEER: transendothelial electrical resistance
Teri: teriflunomide
TGF-β: transforming growth factor β
TLR: toll-like receptor
TNF-α: tumour necrosis factor-alpha
TPA: 12-O-tetradecanoylphorbol-13-acetate
Vpr: viral protein R
Vpu: viral protein U
